# MALDI MSI for a fresh view on atherosclerotic plaque lipids

**DOI:** 10.1007/s00424-021-02654-8

**Published:** 2021-12-20

**Authors:** Anna Worthmann, Alexander Bartelt

**Affiliations:** 1 Department of Biochemistry and Molecular Cell Biology, University Medical Center Hamburg-Eppendorf, Hamburg, Germany; 2 Institute for Cardiovascular Prevention (IPEK), Ludwig-Maximilians-University, Munich, Germany; 3 Institute for Diabetes and Cancer (IDC), Helmholtz Center Munich, Neuherberg, Germany; 4 German Center for Cardiovascular Research, Partner Site Munich Heart Alliance, Munich, Germany; 5 Department of Molecular Metabolism & Sabri Ülker Center for Metabolic Research, Harvard T.H. Chan School of Public Health, Boston, MA USA

**Fig. 1:**
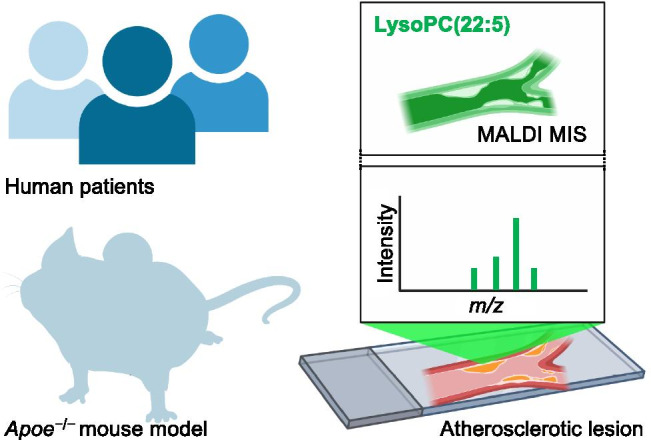
MALDI MIS of atherosclerosis. Human and mouse specimens (atheroprone apolipoprotein E–deficient mice) were compared using matrix-assisted laser desorption/ionization (MALDI) mass spectrometry imaging (MSI). A major novel biomarker discovered was LysoPC(22:5), which was specifically abundant in human and mouse atherosclerotic lesions

Atherosclerosis is an immunometabolic disorder underlying coronary artery disease and stroke, which remain the leading cause of death and morbidity worldwide. As atherogenesis results in the formation of complex lipid-laden lesions, a deep spatio-temporal understanding of lipid metabolism during plaque formation is key to developing new therapeutic approaches. The nature of lipid species in atherosclerotic lesions is very complex, and technological advancement in the field of mass spectrometry (MS)–based detection of lipids (“lipidomics”) has helped to understand the etiology of atherosclerosis better [1]. The new study by Khamehgir-Silz et al. (this issue) now uses matrix-assisted laser desorption/ionization (MALDI) MS imaging (MSI) to investigate atherosclerotic plaque composition in mouse and human plaque specimens (Fig. 1). The main conclusions are that early atherogenesis in apolipoprotein E (apoE)–deficient animals, a common transgenic animal model for hypercholesterolemia and plaque formation [2], was not associated with major changes compared to wild-type controls. Interestingly, lesions from older apoE-deficient animals with advanced atherosclerosis were more similar to human plaques, which were, unsurprisingly, extremely heterogeneous. Several aspects in the study advance the field. The comparison of young vs. older apoE-deficient mice sheds light on lipidomic changes during the progression of atherosclerosis. While previous studies started analyzing human atherosclerotic lesion samples by MALDI MIS [3–6], this investigation directly compared the lipid landscapes between mouse and human lesions with a robust sample number. This means, instead of demonstrating a qualitative validation, the work by Khamehgir-Silz et al. [7] measured the differential lipid composition in quantitative terms, setting a new reference standard for plaque MALDI MIS. Another novel aspect of this work is the improved spatial resolution compared to previous studies [3–6] as Khamehgir-Silz et al. achieved 7 µm for mouse and 5–15 µm for human samples, allowing for cellular resolution on the tissue level. This is important because it provides a map of the specific lipid location intima vs. media vs. adventitia. In the future, it will be critical to combine protein and lipid maps to facilitate the creation of lipidomic landscapes of endothelial cells, smooth muscle cells, or immune cells. Also, Khamehgir-Silz et al. identified new lipid classes that are apparently specific for human plaques, i.e., acyl steryl glucosides, for example, 16:0-Glc-cholesterol or 18:3-Glc-cholesterol, which are potentially advanced glycation end products. Also, LysoPC(22:5) was detected with high confidence in mouse and human plaques. Future studies will have to show how these lipids are formed, what their biological/pathological relevance is, and how they might serve as non-invasive plasma biomarkers for detecting unstable atherosclerotic plaques or atheroregression [8]. A few limitations remain: in MSI, the lipids are directly transferred to the mass spectrometer without any further separation, so that low abundance lipids are not included in the analysis as their signal is suppressed by high abundance lipids. Next, simple acquisition of MS spectra does not allow for the unequivocal identification of lipids (for example, *m*/*z* 532.28 could be LysoPE(18:0) or LysoPC(16:1). Therefore, lipids should be identified by MS^n^ experiments, where the *m*/*z* of interest is repeatedly isolated and fragmented to gain further structural information. In addition, targeted MS employing standards for the subsequent validation of the aforementioned new markers should be performed with spatial information. In conclusion, the study by Khamehgir-Silz et al. represents a methodological advancement for the study of atherosclerosis. It will be exciting to see how much MALDI MSI can contribute to a better understanding of cell-specific lipid dynamics in atherosclerosis. A robust platform for spatio-temporal lipidomics will advance the study of cardiometabolic diseases with strong contributions of aberrant lipid metabolism such as obesity or non-alcoholic fatty liver disease [9].
